# Trend of Lassa fever cases and factors associated with mortality in Liberia, 2016 - 2021: a secondary data analysis

**DOI:** 10.11604/pamj.2024.47.22.42156

**Published:** 2024-01-19

**Authors:** Emmanuel Dwalu, Ralph Weah Jetoh, Bode Ireti Shobayo, Irene Pewu, Fahn Taweh, Himiede Wede Wilson-Sesay, Godwin Etim Akpan, Fulton Shannon, Babalola Obafemi Joseph, Chukwuma David Umeokonkwo, Peter Adewuyi, Maame Amo-Addae, Thomas Knue Nagbe, Julius Gilayeneh, Jane Amanda MaCauley

**Affiliations:** 1 National Public Health Institute of Liberia, Monrovia, Liberia; 2 Liberia Field Epidemiology Training Program, Monrovia, Liberia; 3 African Field Epidemiology Network, Monrovia, Liberia

**Keywords:** Case fatality, confirmed case, endemic, Lassa fever, positivity rate, trend

## Abstract

**Introduction:**

Lassa fever (LF) is endemic in Liberia and is immediately reportable. Suspected cases are confirmed at the National Public Health Reference Laboratory. However, there is limited information on the trend and factors associated with mortality. We described the epidemiological characteristics of LF cases and determined factors associated with mortality in Liberia from 2016 to 2021.

**Methods:**

we reviewed 867 case-based LF surveillance data from 2016 to 2021 obtained from the National Public Health Institute of Liberia (NPHIL). The cases that met the suspected LF case definition were tested with RT-PCR. Using Epi Info 7.2.5.0. We conducted univariate, bivariate, and multivariate and analysis. We calculated frequencies, proportions. Positivity rate, case fatality rate, and factors associated with LF mortality using chi-square statistics and logistics regression at 5% level of significance.

**Results:**

eighty-five percent (737/867) of the suspected cases were tested and 26.0% (192/737) were confirmed LF positive. The median age of confirmed LF cases was 21(IQR: 12-34) years. Age 10-19 years accounted for 24.5% (47/192) and females 54.2% (104/192). Bong 33.9% (65/192), Grand Bassa 31.8% (61/192), and Nimba counties, 21.9% (42/192) accounted for most of the cases. The median duration from symptom onset to hospital admission was 6 (IQR: 3-9) days. A majority, 66% (126/192) of the cases were reported during the dry season (October-March) and annual incidence was highest at 12 cases per 1,000,000 population in 2019 and 2020. The overall case fatality rate was 44.8%. Non-endemic counties, Margibi, 77.8% and Montserrado, 66.7% accounted for the highest case fatality rate (CFR), while 2018, 66.7% and 2021, 60.0% recorded the highest CFR during the period. Age ≥30 years (aOR=2.1,95% CI: 1.08-4.11, p=0.027) and residing in Grand Bassa County (aOR=0.3, 95% CI: 0.13-0.73, p=0.007) were associated with LF mortality.

**Conclusion:**

Lassa fever was endemic in three of the fifteen counties of Liberia, case fatality rate remained generally high and widely varied. The high fatality of LF has been reported to the NPHIL and is currently being further investigated. There is a need to continuously train healthcare workers, especially in non-endemic counties to improve the LF treatment outcome.

## Introduction

Lassa fever (LF) is a zoonotic disease with multimammate rat (*Mastomys natalensis*) as the animal reservoir [1,2]. This multimammate rat has been described as one of the most extensive rodent species in West Africa and is frequently found in rural homes [3]. The primary mode of spread is from rodent to man through contact with rodent excreta or urine in food or during hunting and processing of rats for consumption and has an incubation period ranging from 6-21 days [3]. The disease usually has gradual signs and symptoms, beginning with fever, general weakness, and malaise; and after a few days, headache, sore throat, muscle pain, chest pain, nausea, vomiting, diarrhea, cough, and abdominal pain. Where cases are severe, it may develop facial swelling, fluid in the lung cavity, bleeding from the mouth, nose, vagina, or gastrointestinal tract, and low blood pressure and about 80% of people who become infected have no symptoms [3,4]. In the West African region, there has been tremendous rise in Lassa fever cases [5] and deaths over the years since the first case was recorded in Lassa Town, Nigeria [6].

The disease is endemic in Nigeria, Sierra Leone, Guinea, and Liberia with high burden reported annually [7,8]. The overall case-fatality rate is 1% but 15% among hospitalized. The number of Lassa virus infections per year in West Africa is estimated at 100,000 to 300,000, with approximately 5,000 deaths. Unfortunately, such estimates are crude, because surveillance for cases of the disease is not uniformly performed. In some areas of Sierra Leone, it is known that 10%-16% of people admitted to hospitals every year have Lassa fever, which indicates the serious impact of the disease on the population of this region [5]. The disease occurs in all age groups and both sexes. Persons at greatest risk are those living in rural areas where Mastomys are usually found, especially in communities with poor sanitation or crowded living conditions. Health workers are at risk if caring for Lassa fever patients in the absence of proper barrier nursing and infection prevention and control practices. Early supportive care with rehydration and symptomatic treatment improves survival. Lassa fever is endemic in Liberia with a continuous increase in the number of confirmed cases and deaths [2,9]. It is an immediately reportable disease under surveillance within the Integrated Disease Surveillance and Response system [10].

There have been suspected Lassa fever cases reported in Liberia since 2016 [2] but seven (7) of the fifteen (15) counties (Bong, Grand Bassa Margibi, Montserrado, Nimba, Lofa, and Grand Kru) have recorded confirmed cases [11]. Historically, it has been recorded that the disease is endemic in six counties: Bong, Grand Bassa Margibi, Montserrado, Nimba, and Lofa [12,13]. Before 2017, Bong, Montserrado, Nimba, and Lofa were the original endemic counties but Grand Bassa, Margibi, and Grand Kru Counties began reporting cases in 2017 (Yota, 2019 and NPHIL, 2020). To date, seasonal outbreaks of Lassa fever have been consistently reported in Liberia with an increase in confirmed cases and deaths [11,12]. Historical trends show that infection with the virus has been seasonal in Liberia, especially during the dry season in the early and later parts of the year and fatality is generally high within the subregion [2] (Yota, 2019 and NPHIL, 2020) [11]. Despite some progress made in the diagnosis and treatment of Lassa fever in Liberia, there is limited information on the trend and factors associated with Lassa fever mortality in Liberia. We described the epidemiological characteristics of Lassa fever cases and determined factors associated with Lassa fever mortality in Liberia from 2016 to 2021.

## Methods

**Study setting:** Liberia is a sub-Saharan nation with an approximated population of 5,057,681. Liberia is divided into 15 political subdivisions, called counties. These counties are subdivided into 93 health districts with 850 health facilities and several communities. Liberia is implementing the Integrated Disease Surveillance and Response Strategy as disease surveillance system. As of June 2021, Liberia has a total of 21 priority diseases, conditions, events under surveillance including Lassa fever. There are five tiers of information flow within the surveillance system including community, health facility, district, county, and national. Each of the tiers have staff including national supervisors, county surveillance officers (CSOs), district surveillance officers (DSOs), health facility surveillance focal persons (HFSFPs), and community health services supervisors (CHSS), community health assistants (CHAs), and community health volunteers (CHVs). The country has two distinct seasons: rainy and dry season. The rainy season runs from May to October, while the dry season runs from October to April. Cases of priority diseases including Lassa fever are detected from the community and health facility and reported to district, county, and national levels. Lassa fever is one of the priority diseases endemic in Liberia with increase in cases and outbreaks [9,11]. There are decentralized treatment facilities for Lassa fever in Liberia including Redemption Hospital (Montserrado County), Phebe Hospital (Bong County), Liberia Agriculture Company Hospital (Grand Bassa County), Ghanta United Methodist Hospital (Nimba County), CH Rennie Hospital (Margibi County), and Tellewoyan (Lofa County). Specimen for suspected cases are collected from health facilities and transported to the National Public Health Reference Laboratory for confirmation using the Real-Time Polymerase Chain Reaction Test (RT-PCR) [10].

**Study design:** this study was a secondary data analysis involving records of all Lassa fever cases reported in Liberia from 2016-2021.

**Study participants, data source, flow and cleaning procedures:** we reviewed 867 case-based Lassa fever surveillance data from 2016 to 202. We obtained data from the weekly surveillance reports submitted by counties to the National Public Health Institute of Liberia. According to Liberia´s Integrated Disease Surveillance and Response Technical Guidelines and Lassa fever contingency plan (NPHIL, 2020) [10], the case definitions for Lassa fever are described below

**Suspected case:** patient with fever for 2-21 days with a measured temperature of 38°C or more with one or more of the following symptoms: vomiting, diarrhea, sore throat, myalgia (muscle pain), generalized body weakness, abdominal pain, abdominal bleeding. A patient who has not responded to antimalaria treatment for other infectious causes of fever within 48-72 hours.

**Probable case:** a suspected case who has one or more of the following complications: hearing loss, facial or neck swelling, seizures, restlessness, confusion, hypotension, abdominal bleeding

**Confirmed case:** a suspected or probable case that is laboratory confirmed (positive IgM antibody, using real-time Polymerase Chain Reaction or virus isolation, or epidemiologically linked to a laboratory confirmed case. Data flows form community, health facility and reported to district, county, and national level for use to inform intervention. We reviewed/crosschecked data collected for accuracy or any missing variables before analysis. The data was cleaned using MS Excel 2016 and exported to Epi Info 7 for analysis.

**Variables of interest:** variables in the dataset were age, sex, county, district, health facility, site of detection, date of onset, date of admission, reporting year, month, and date of specimen collection, date tested, Lab results, and outcome. However, we selected age, sex, county, district, health facility, site of detection, date of onset, date of admission, reporting year, month, and outcome for analysis. The variables were categorized into outcome and exposure. The outcome variables were alive and dead while age, sex, county, district, health facility, site of detection, site of detection, duration from admission were the exposure variables.

**Data analysis:** after cleaning our data, we analyzed using Epi info version 7.2.5.0. We conducted univariate, bivariate, and multivariate analyses. For univariate analysis, we calculated frequencies, proportions for age, sex, county, district, and health facility, site of detection and duration from onset to admission. We categorized age and duration from onset to admission into groups and calculated median age and duration as well as interquartile range. Additionally, we analyzed by month and year over the study period to show the trend, endemicity, and the seasonality. We examined the association between Lassa fever fatality and age, sex, county, district, and health facility, site of detection and duration from onset to admission using chi-square (χ^2^) at 95% confidence interval. Associations with P values < 0.25 at bivariate level were entered into multiple logistic model. Results were presented in tables, charts, and maps.

**Ethical consideration:** approval for data use was granted by the National Public Health Institute of Liberia. We maintained confidentiality by delinking identifiers from the results and report.

## Results

**Figure 1:**
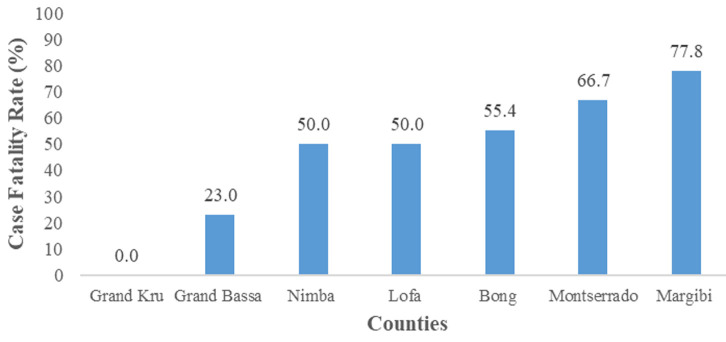
Lassa fever case fatality rate (CFR) by county, Liberia, 2016 - 2021

**Figure 2:**
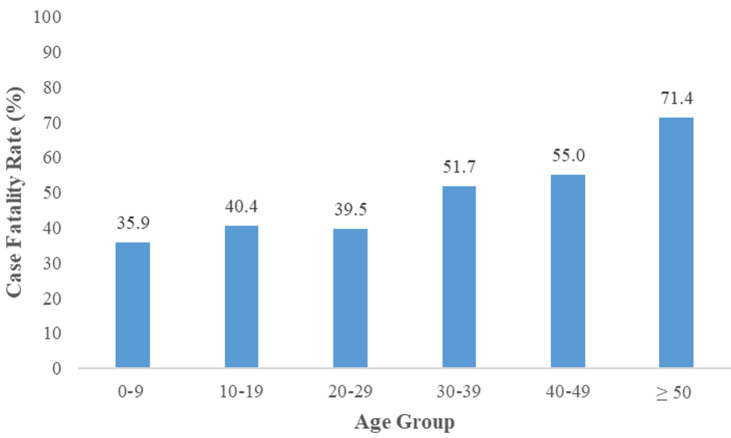
Lassa fever case fatality rate (CFR) by age, Liberia, 2016 - 2021

A total of 867 suspected cases of Lassa fever were reported in Liberia for the study period. Of the 867 suspected cases, 85.0% (737/867) were tested and 26.1% (192/737) confirmed Lassa fever positive. The overall positivity rate (PR) was 26.1% (192/737) but varied from 0% to 31.8%. Grand Bassa County accounted for the highest positivity rate, 31.8% (61/192) followed by Bong, 30.2% (65/215) and Nimba, 28.4% (42/148) while Montserrado County accounted for the least, 12.0% (9/75) (Table 1). The median age of the cases was 21 (interquartile range (IQR): 12-34) years. The highest cases were recorded in age 10-19 yrs, 24.5% (47/192), followed by 20-29 yrs, 22.4% (43/192), while age ≥ 50 accounted for the least, 7.3% (14/192). About half of the cases were females, 54.2% (104/192). Bong county accounted for the majority of the cases, 33.9% (65/192), followed by Grand Bassa 31.8% (61/192), while Grand Kru accounted for the least, 1% (2/19). Among health districts, District 3 A andB accounted for 30.2% (58/192), followed by Suakoko District 29.2% (56/192). Tertiary facility accounted for 88.0% (169/192) and majority of the cases were alive, 55% (n=106/192). The median duration from onset to admission was 6 (IQR: 3-9) days, 0-6days accounted for 55.7% (107/192), and the majority of the cases, 94% (181/192) were detected at the health facility (Table 2). Regarding the case fatality rate (CFR) by county and age, the overall CFR was 45% (86/192). Margibi County recorded the highest CFR, 78% (7/9), followed by Montserrado, 67% (6/9). Bong, 55% (36/65) and Grand Bassa, 23% (14/61) recorded the highest confirmed cases but lower CFRs (Figure 1). Age≥50 yrs accounted for the highest fatality, 71% (10/14), followed by 40-49yrs, 55% (11/20), and 30-39 yrs, 52% (15/29), while 0-9yrs accounted for the least, 36% 14/39) (Figure 2).

**Table 1:** Lassa fever positivity rate by County, Liberia, 2016-2021 (n=867)

County	Suspected	Tested	Confirmed	Positivity rate (%)
Grand Bassa	207	192	61	31.8
Bong	254	215	65	30.2
Nimba	190	148	42	28.4
Margibi	53	43	9	20.9
Lofa	24	21	4	19.0
Grand Kru	18	13	2	15.4
Montserrado	83	75	9	12.0
Maryland	12	11	0	0.0
Gbarpolu	7	6	0	0.0
Bomi	4	4	0	0.0
Sinoe	5	3	0	0.0
Grand Cape mount	2	2	0	0.0
Rivercess	3	2	0	0.0
River gee	4	1	0	0.0
Grand Gedeh	1	1	0	0.0
**Total**	867	737	192	26.1

**Table 2:** distribution of confirmed Lassa fever cases, Liberia, 2016 -2021

Characteristics	Frequency (n=192)	Percent (%)
**Age (Years)**		
0-9	39	20.3
10-19	47	24.5
20-29	43	22.4
30-39	29	15.1
40-49	20	10.4
≥50	14	7.3
**Median age**	**21 (IQR:12-34**) **years**	
**Sex**		
Female	104	54.2
Male	88	45.8
**County of residence**		
Bong	65	33.9
Grand Bassa	61	31.8
Nimba	42	21.9
Margibi	9	4.7
Montserrado	9	4.7
Lofa	4	2.1
Grand Kru	2	1.0
**Reporting district**		
District # 3A&B	58	30.2
Suakoko	56	29.2
Sanniquellie-Mah	35	18.2
Kakata	8	4.2
Commonwealth	6	3.1
Jorquelleh	6	3.1
Saclepea-Mah	6	3.1
Central Monrovia	4	2.1
*Others	13	6.8
**Facility type**		
Tertiary	169	88.0
Secondary	23	12.0
**Outcome**		
Alive	106	55.2
Dead	86	44.8
**Duration from onset to admission**		
0 – 6	107	55.7
7-13	61	31.8
14≥	24	12.5
**Median duration (days)**	**6 (IQR: 3-9)**	100.0
**Site of detection**		
Health facility	181	94.3
Community	11	5.7
**Endemicity**		
Endemic	168	87.5
Non-endemic	24	12.5
**Seasonality**		
Dry	138	71.9
Rainy	54	28.1
*Others; districts reporting 1 or 2 cases		

**Figure 3:**
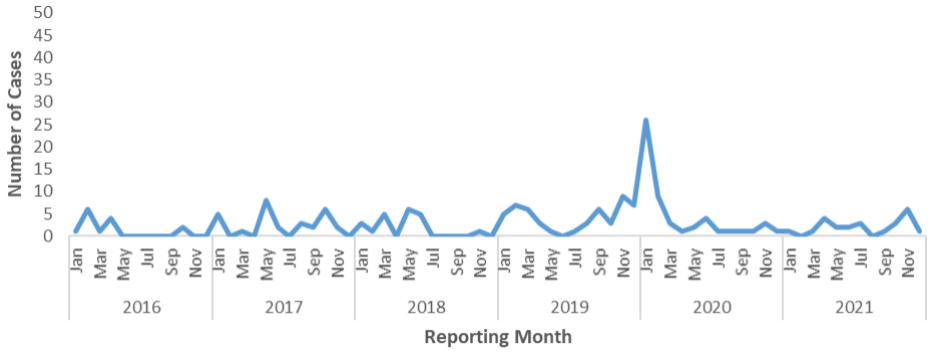
trend of Lassa fever cases by reporting month and year, Liberia, 2016 - 2021

**Figure 4:**
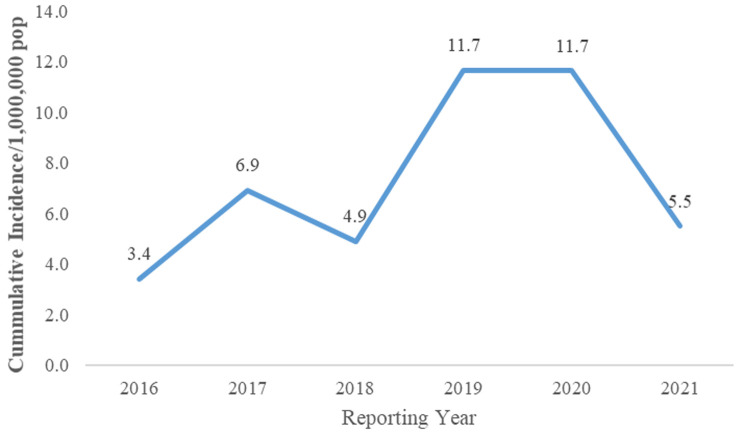
cumulative incidence of Lassa fever cases by year, Liberia, 2016 - 2021

**Trend of Lassa fever cases by Reporting Month and Year, Liberia, 2016 - 2021:** majority of the cases, 66% (126/192) of the cases, were reported during the dry season (October-March) (Figure 3). For cumulative incidence, the years 2019 and 2020 recorded the highest incidence over the period respectively, 12/1,000,000 population (Figure 4). Lassa fever is endemic in three (3) of the fifteen (15) counties of Liberia, but confirmed cases have been reported in seven (7) of the fifteen (15) counties from 2016-2021. Of the 7 counties, Bong, Grand Bassa, and Nimba Counties reported Lassa fever cases consistently in 5 of the 6 years studied, while Montserrado, Margibi, Grand Kru, and Lofa reported sporadic cases (Table 3). We found significant associations between age (≥30 yrs (cOR=2.1, 95% CI=1.1 - 1.9, p = 0.016) and Lassa fever mortality. The associations between tertiary facility (cOR=1.3, 95% CI=0.5-3.1, p=0.560) and non-endemic county (cOR=2.2, 95% CI=0.9-5.4, p=0.062) were not significant. For county of residence, Grand Bassa County (cOR=0.2, 95% CI=0.1- 0.4, p<0.001) was less likely associated with Lassa fever mortality. At multivariate level, we found that age (≥30 yrs) (aOR=2.1, 95% CI=1.0 - 4.1, p=0.027) is a factor associated with LF mortality while Lassa fever cases residing in Grand Bassa County (aOR=0.2, 95% CI=0.1-0.7, p=0.007) have better outcome compared to others (Table 4).

**Table 3:** endemicity of Lassa fever in Liberia, 2016-2021

Reporting County	2016	2017	2018	2019	2020	2021	Total
Bong	8	7	5	17	19	9	65
Grand Bassa	0	9	2	18	23	9	61
Nimba	4	9	9	9	5	6	42
Montserrado	1	0	2	2	3	1	9
Margibi	0	2	3	3	1	0	9
Grand Kru	0	1	0	1	0	0	2
Lofa	1	1	0	1	1	0	4
**Total**	14	29	21	51	52	25	192

**Table 4:** factors associated with Lassa fever mortality, Liberia, 2016-2021

Characteristics	Outcome		cPOR (95% CI)	P value	aPOR (95% CI)	P-value
Age (years)	Dead (%)	Alive (%)				
≥ 31 years	31 (56)	24 (44)	*1.8 (1.00-3.13)	0.041	2.3 (1.12-4.57)	0.023
< 31years	55 (40)	82 (60)	1			
**Sex**						
Female	46 (44)	58 (56)	1.0 (0.54-1.68)	0.865	1.3 (0.67-2.39)	0.460
Male	40 (45)	48 (55)	1			
**County of residence**						
Bong	36 (55)	29 (45)	*2.0 (1.04-3.50)	0.035	1.2 (0.59 -2.54)	0.587
Others	50 (39)	77 (61)	1			
**Managing health facility**						
LAC hospital	13 (22)	47 (78)	*0.2 (0.11-0.45)	0.001	0.2 (0.10-0.49)	<0.001
Others	73 (55)	59 (45)	1			
**Site of detection**						
Health facility	80 (93)	101 (95)	0.7 (0.19-2.24)	0.050	0.6 (0.17-2.37)	0.501
Community	6 (7)	5 (5)	1			
**Onset to date seen**						
≥ 7 days	36 (42)	49 (58)	0.8 (0.47-1.49)	0.545	0.9 (0.48 - 1.69)	0.515
< 7 days	50 (47)	57 (53)	1			
**Seasonality**						
Dry season	61 (44)	77 (56)	1.0 (0.50-1.73)	0.793	1.1 (0.52-2.15)	0.880
Rainy season	25 (46)	29 (54)	1			
**Endemicity**						
Non-endemic	15(65)	9(37)	*2.3 (1.00-5.50)		3.2(1.26-8.24)	0.01
Endemic	71(42)	97(57)				

* = significant p-values/associations

## Discussion

This study described the epidemiological characteristics of Lassa fever cases and determined factors associated with Lassa fever mortality in Liberia from 2016 to 2021. The findings showed that Lassa fever is endemic in three of the fifteen counties of Liberia instead of the traditionally mentioned six endemic counties, there is high fatality amongst cases, especially in non-endemic counties, older age groups, and females, the overall progressive increase in the incidence of cases especially in 2019 and 2020, majority of the cases are reported during the dry season, age and county of residence are factors associated with Lassa fever mortality. For endemicity, Liberia is one of the countries in the Lassa fever belt within the West African region with high disease burden and mortality (Sierra Leone, Nigeria, Guinea, and Liberia) [7,8].

There have been suspected Lassa fever cases reported in Liberia since 2016 with seven (7) of the fifteen (15) counties including Bong, Grand Bassa Margibi, Montserrado, Nimba, Lofa, and Grand Kru [11]. Of these, three (3) counties including Bong, Grand Bassa, and Nimba) are endemic to Lassa fever (they have consistently reported confirmed cases over the last five years), while the rest of the other four (4) counties (Margibi, Montserrado, Lofa, and Grand Kru) have reported sporadic cases (Yota, 2019 and NPHIL, 2020) [11,12,14]. This finding could be likely because the three endemic counties identified (Bong, Grand Bassa, and Nimba) are located in rural parts of Liberia where inhabitants are involved with farming and sometimes hunting and consuming rats as well as not observing environmental sanitation, thereby exposing them to the Lassa virus through contact with the faces, blood, urine, etc during preparation. This finding is similar to a study by WHO which showed that Liberia is endemic to Lassa fever (WHO, 2018) [15]; consistent with a study in Sierra Leone which showed that LF is highly endemic in Sierra Leone, especially in the Eastern region [16], and a study in Nigeria which also showed that the majority of the cases were recorded in two states (Edo and Ondo) in 2020 but differs in that it reported the spread of Lassa fever cases from 19 states in 2017 to 32 states and two Federal Capital Territories in 20 [1].

The finding also is similar to the study in Guinea which shows that Lassa fever is a disease of rural areas influenced by the composition of the rodent population [17]. The high fatality in non-endemic counties and older age females could be likely because of the asymptomatic presentation of Lassa fever cases and clinicians in non-endemic counties have low caseload, which limits their ability to quickly suspect and experience in managing Lassa fever cases compared to those in endemic counties. Additionally, fatality among older age females could be likely due to female involvement in providing care for others including children, be it at home or elsewhere, which sometimes exposes them to the virus through contact with excreta, urine, or faeces of the rodents during the process. These findings are similar to a study by WHO which showed high (45.4%) case fatality among cases (WHO, 2018); a study in Sierra Leone which found that a significantly higher proportion of females than males had Lassa fever [18], similar to the study in Nigeria which found high case fatality among older age groups but differs in that very young age groups were also in majority [19]. Similarly, the study's high case fatality of Lassa fever was also recorded during a hospital epidemic in a study in Zorzor, Lofa Lofa County, Liberia [20].

In this study, the majority of the cases were reported in 2019 and 2020, especially during the dry season in Liberia (October-March). The increase in cases could be likely because of the improved surveillance system following updating of Liberia´s Integrated Disease Surveillance and Response strategy from 2^nd^ to 3^rd^ edition which increased case detection and reporting among surveillance actors at the sub-national level. Similarly, by [2] the cases peaked in 2019 and 2020. The seasonal increase in cases could be likely because the rodents breed especially during the dry season, thereby increasing their population in the communities and homes which puts residents at risk of infection with the Lassa fever virus. The seasonal pattern revealed by our study is consistent with the study in Nigeria where cases were seen to peak in the dry season (December-March) each year [21]. However, this study differs from a study conducted in Sierra Leone, which showed that Lassa fever infection occurs during the dry season in March as well as during the rainy season in June and July [22].

Findings from this study showed that the majority of the cases are presented at the health facility for treatment, within one to six days from onset to admission but high case fatality rate. This could likely be due to the sometimes-asymptomatic nature or nonspecific early signs and symptoms of Lassa fever, which sometimes delay identification or diagnosis and result in worse outcomes when the infection is severe. This finding is similar to the study conducted in Nigeria among pregnant women, which showed high fatality due to late care seeking and delay in identification or diagnosis of Lassa fever among patients (Okogbenin *et al*. 2019) [23]. Our findings showed that age is a factor associated with Lassa fever mortality and cases residing in Grand Bassa County have better treatment outcomes compared to others. This good outcome of Lassa fever patients in Grand Bassa County could likely be so because Grand Bassa County is one of the Lassa fever endemic counties in Liberia that has the Liberia Agricultural Hospital as the major treatment facility for cases. Clinicians at this facility are familiar with the case definition and timely detecting Lassa fever and initiating treatment, thereby resulting in better outcomes. This study is contrary to findings from a study conducted in a dedicated treatment facility in Nigeria, which found a strong association between acute kidney injury and Lassa fever mortality instead of age [24]. In convergence with this study, another study in Nigeria showed that the association between age and Lassa fever mortality was statistically significant (Tobin 2014) *et al*. [25].

**Limitations:** variables such as ribavirin administration to cases and possible environmental practices exposing cases could not be accessed in the dataset for analysis. However, the records reviewed and variables analysed were sufficient to validate the findings of this study.

## Conclusion

We report that Lassa fever is endemic in three of the fifteen counties of Liberia instead of the traditionally mentioned six endemic counties. There is high fatality amongst cases in non-endemic counties, especially older age females. There is a progressive increase in incidence of cases especially in 2019 and 2020 with majority of the cases reported during the dry season. Age was identified as a factor associated with Lf mortality and residing in Grand Bassa County had better outcome compared to others. There is a need to continuously train healthcare workers especially in non-endemic counties to improve the LF treatment outcome.

### What is known about this topic

- *Historically, it has been recorded that Lassa fever is endemic in Liberia;*
- *Also, it is known that Lassa fever is a seasonal disease in Liberia, reported during the dry season*.

### What this study adds

- *This study has provided a clear guidance to the national surveillance system through the National Public Health Institute of Liberia on the actual endemicity of Lassa fever in Liberia other then what was historically known;*
- *Additionally, this paper presents a five-year trend analysis that showed the burden of Lassa fever cases in Liberia by person, place, time, as well as factors associated with mortality;*
- *This study has also informed the surveillance system that there have been Lassa fever cases reported in counties other than endemic counties and counties endemic to Lassa fever have better outcome compared to non-endemic counties*.
